# Mapping the risk of infections in patients with multiple sclerosis: A multi-database study in the United Kingdom Clinical Practice Research Datalink GOLD and Aurum

**DOI:** 10.1177/13524585221094218

**Published:** 2022-05-14

**Authors:** Melissa WY Leung, Marloes T Bazelier, Patrick C Souverein, Bernard MJ Uitdehaag, Olaf H Klungel, Hubert GM Leufkens, Romin Pajouheshnia

**Affiliations:** Division of Pharmacoepidemiology & Clinical Pharmacology, Utrecht Institute for Pharmaceutical Sciences (UIPS), Department of Pharmaceutical Sciences, Faculty of Science, Utrecht University, Utrecht, The Netherlands; Division of Pharmacoepidemiology & Clinical Pharmacology, Utrecht Institute for Pharmaceutical Sciences (UIPS), Department of Pharmaceutical Sciences, Faculty of Science, Utrecht University, Utrecht, The Netherlands; Division of Pharmacoepidemiology & Clinical Pharmacology, Utrecht Institute for Pharmaceutical Sciences (UIPS), Department of Pharmaceutical Sciences, Faculty of Science, Utrecht University, Utrecht, The Netherlands; Department of Neurology, Amsterdam Neuroscience, VUmc MS Center Amsterdam, VU University Medical Center, Amsterdam, The Netherlands; Division of Pharmacoepidemiology & Clinical Pharmacology, Utrecht Institute for Pharmaceutical Sciences (UIPS), Department of Pharmaceutical Sciences, Faculty of Science, Utrecht University, Utrecht, The Netherlands; Division of Pharmacoepidemiology & Clinical Pharmacology, Utrecht Institute for Pharmaceutical Sciences (UIPS), Department of Pharmaceutical Sciences, Faculty of Science, Utrecht University, Utrecht, The Netherlands; Division of Pharmacoepidemiology & Clinical Pharmacology, Utrecht Institute for Pharmaceutical Sciences (UIPS), Department of Pharmaceutical Sciences, Faculty of Science, Utrecht University, Utrecht, The Netherlands

**Keywords:** Multiple sclerosis infections, multiple outcomes multi-database study, United Kingdom Clinical Practice Research Datalink GOLD and Aurum, urinary tract infection

## Abstract

**Background:**

People with multiple sclerosis (pwMS) have an increased risk of infections; risk factors include underlying disease, physical impairment and use of some disease-modifying treatments.

**Objective:**

To quantify changes in population-level infection rates among pwMS and compare these to the general population and people with rheumatoid arthritis (pwRA), and identify patient characteristics predictive of infections after MS diagnosis.

**Methods:**

We conducted a multi-database study using data on 23,226 people with MS diagnosis from the UK Clinical Practice Research Datalink Aurum and GOLD (January 2000–December 2020). PwMS were matched to MS-free controls and pwRA. We calculated infection rates, and estimated incidence rate ratios (IRR) and 95% confidence intervals (CI) of predictors for infections ⩽ 5 years after MS diagnosis using Poisson regression.

**Results:**

Among pwMS, overall infection rates remained stable – 1.51-fold (1.49–1.52) that in MS-free controls and 0.87-fold (0.86–0.88) that in pwRA – although urinary tract infection rate per 1000 person-years increased from 98.7 (96.1–101) (2000–2010) to 136 (134–138) (2011–2020). Recent infection before MS diagnosis was most predictive of infections (1 infection: IRR 1.92 (1.86–1.97); ⩾2 infections: IRR 3.00 (2.89–3.10)).

**Conclusion:**

The population-level elevated risk of infection among pwMS has remained stable despite the introduction of disease-modifying treatments.

## Introduction

People with multiple sclerosis (pwMS) have a higher risk of mild and severe infections than the general population, which is already apparent in the prodromal phase of MS.^1–4^ MS disease progression can cause dysfunction of the urinary, respiratory and gastrointestinal tracts leading to increased susceptibility to infection, while reduced mobility may also increase the risk of infection of the respiratory tract, urinary tract and skin and subcutaneous tissue. PwMS are 2.0–2.4 times as likely to be hospitalized for infection as the general population,^5–7^ and visit the physician for infection 1.4 times as frequently.^6^

Aside from clinical characteristics, some disease-modifying treatments (DMTs) alter the risk of infections through their immunomodulatory or immunosuppressive effects. Concerns over the safety profile of some biologics have led to recommendations that the increased risk of infections should factor into the benefit-risk assessment of MS therapies.^8^ This also holds for biologics used for the treatment of rheumatoid arthritis (RA), another immune-mediated disease in which disease activity and patient characteristics are associated with increased infection risk.^9,10^

While evidence of the effects of DMTs on infection risk is accumulating, the MS population has continued to evolve. Earlier diagnosis^11^ and an ageing, multi-comorbid population^12^ make it challenging to disentangle the increased infection risk due to treatment from other risk factors. In addition, it is unclear whether the changing treatment availability has impacted infection risk after MS diagnosis, and which pwMS are at the greatest risk.

For more tailored prevention and care, pwMS’ risk of infections needs to be understood as a result of the interplay between patient characteristics, functional limitations and treatments. To potentially untangle some of these factors, infections were studied in comparison to two different control groups: the general population and people with RA (pwRA), who had been identified as having a somewhat similar type of disease, including the availability of treatment with biologics. Therefore, we aimed to assess whether infection rates among pwMS have changed over the past two decades, compared with the general population and pwRA. Second, we aimed to determine infection rates before and after MS diagnosis and compare these to the general population and pwRA. Third, we aimed to identify patient characteristics at MS diagnosis that predict a higher infection rate and assess whether these have changed over time.

## Materials and methods

### Data source and data collection

Data for this retrospective cohort study were collected from the United Kingdom (UK) Clinical Practice Research Datalink (CPRD) GOLD and Aurum databases, which contain anonymized electronic healthcare records from routine care by general practitioners (GPs) in the UK, capturing diagnoses made in general practice and hospital, as well as GP prescriptions.^13,14^ The active people in CPRD Gold covered approximately 6.9% of the UK population in July 2013, compared with approximately 13% coverage of the English population in September 2018 by active people in CPRD Aurum. People were excluded from the CPRD GOLD dataset if they were included in the Aurum dataset (*n* = 2085 pwMS excluded).

### Participants

**Figure 1.**
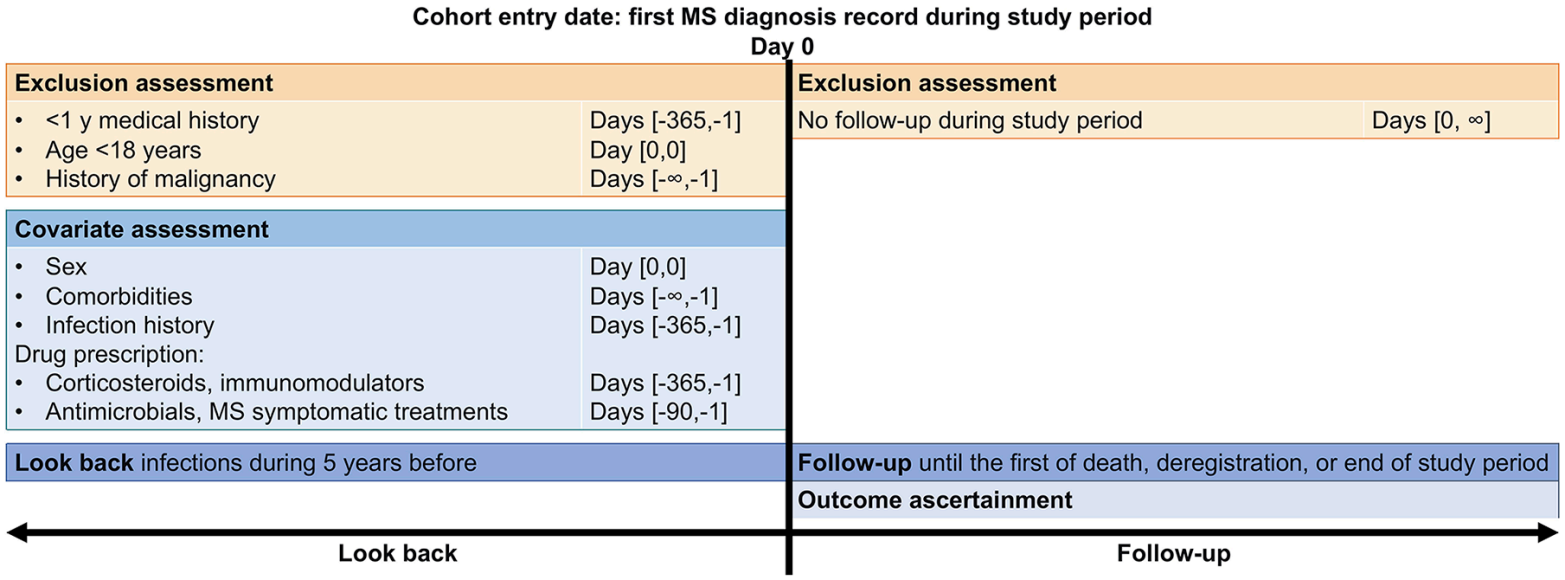
Graphical depiction of the inclusion of people with multiple sclerosis.

Inclusion criteria for pwMS were: the first record of MS diagnosis (index date) during the study period (1 January 2000–31 December 2020), the first record of MS diagnosis after practice’s up-to-standard date (CPRD GOLD only), ⩾ 1 year of medical history, no history of malignancy and age ⩾ 18 years at index date. Follow-up started on the index date and ended at whichever came first: transfer out of GP practice, death, practice’s last collection date, or end of the study period. The study design is depicted graphically in Figure 1.

Within GOLD and Aurum practices, each pwMS was matched to up to two controls from the general population and up to two pwRA. RA diagnosis was ascertained through an algorithm.^15^ Matching was conducted on age (±2 years), index date (±2 years), GP practice and sex. Controls were excluded if they had a record of a demyelinating disorder before the index date. The same assessment windows were applied to controls as to pwMS (Figure 1).

The study protocol (number 20_007) was approved by the CPRD Independent Scientific Advisory Committee (ISAC).

### Outcomes

The main outcome was infection of one of five major types: gastrointestinal tract infection (GTI), urinary tract infection (UTI), skin and subcutaneous tissue infections (SSTI), respiratory tract infection (RTI) and sepsis.^5^ The duration of one episode of infection was assumed to be 28 days.^16,17^ Sepsis was ascertained through diagnosis codes; other types of infections were ascertained through diagnosis codes and symptom codes combined with antimicrobial prescriptions. Code lists and algorithms for infection ascertainment were based on the literature and checked by a medical doctor.^15–24^ Details of the algorithms for the ascertainment of each infection type are available in the Supplemental methods; code lists of medical codes are available online.

### Predictors of infection

Sex, age, recent infection, total number of comorbidities, recent lymphocyte or neutrophil count and recent drug prescriptions were assessed at index date and included as candidate predictor variables of infection. Recent infection comprised the number of episodes of infection of any of the five major types ⩽ 12 months before index date. Comorbidities were ascertained through diagnosis codes recorded ever before index date. They included: diabetes mellitus, chronic lung disease (chronic obstructive pulmonary disease and asthma), inflammatory bowel disease, RA, psoriasis, kidney disease (chronic kidney disease, nephritis, renal hypertensive disease and cystic kidney disease) and cardiovascular risk factor (hypertension, hyperlipidaemia, myocardial infarction, stenting or coronary artery bypass, arrhythmia, valvular disease, heart murmurs, cardiomegaly and congestive heart failure). Recent drug prescriptions included: (1) antimicrobial prescriptions ⩽ 12 months before index date, (2) immunomodulatory drugs (number of unique British National Formulary (BNF) chapter codes) prescribed ⩽ 3 months before index date, (3) symptomatic drugs (number of unique BNF chapter codes) prescribed ⩽ 12 months before index date and (4) number of corticosteroid prescriptions ⩽ 3 months before index date. The antimicrobials included the products listed in the UK National Institute of Health Care and Excellence (NICE) Summary of antimicrobial prescribing guidance – managing common infections.^24^ The immunomodulatory treatments included all disease-modifying antirheumatic treatments, anti-inflammatory treatments, hydroxycarbamide, interferon products, melphalan, mercaptopurine and corticosteroids^25^ other than those used to treat MS relapses, with ⩾ 100 recorded prescriptions in CPRD Gold. The symptomatic drugs included products to treat migraine, sexual dysfunction, dystonia, neuropathic pain, tremor, epilepsy, enuresis, skeletal muscle relaxants, benzodiazepines and anti-depressant drugs.^26^ The corticosteroids included only acute treatments for relapses, as identified in cooperation with a neurologist.

### Statistical methods

Descriptive statistics included median and interquartile range (IQR) of follow-up time and age at index date, and percentages for the categorical variables.

Infection rates per 1000 person-years (PY) were calculated with Poisson exact 95% confidence interval (CI) per calendar year and cohort (pwMS/general population/pwRA), for any of the five types and per type. The trend of infection rates over time was assessed through quasi-Poisson models with the number of infections in each calendar year as the dependent variable, log(PY) as the offset variable and the number of years since study start as the independent variable.^27^ This analysis was conducted overall and stratified by 10-year age groups, sex and infection during the year before index date.

Rates of RTI, SSTI and UTI were calculated from 5 years before to 5 years after index date. Infection rate ratios (IRRs) with Wald 95% CIs were calculated per type of infection by taking the ratio of the infection rate during the 5 years post-index date and the infection rate during the 5 years pre-index date. This analysis was conducted using data from the subgroups of people with ⩾ 5 years of uninterrupted observations before index date and stratified by calendar period: 2000–2010 or 2011–2020, the second half of the study period and when the majority of currently available DMTs were authorized.

Predictors of infection after MS diagnosis were identified using data from a subgroup of pwMS with ⩾ 5 years of follow-up. A multivariable Poisson regression model was derived through backward selection^28^ with the conservative stopping rule of *p* < 0.05. This analysis was stratified by calendar period: 2000–2010 or 2011–2020.

All analyses were conducted on data from CPRD GOLD and Aurum, and the results were pooled using Rubin’s rules.^29^

### Sensitivity analyses

In one sensitivity analysis, the duration of an episode of infection was defined as 60 days to investigate possible detection bias in the results. In another sensitivity analysis, to identify predictors of infection after MS diagnosis, a multiple-events survival analysis was conducted using all outcome data in the Andersen–Gill model, to allow for censoring and take into account the within-subject correlation between event times.^30^

## Results

### Participants

**Figure 2.**
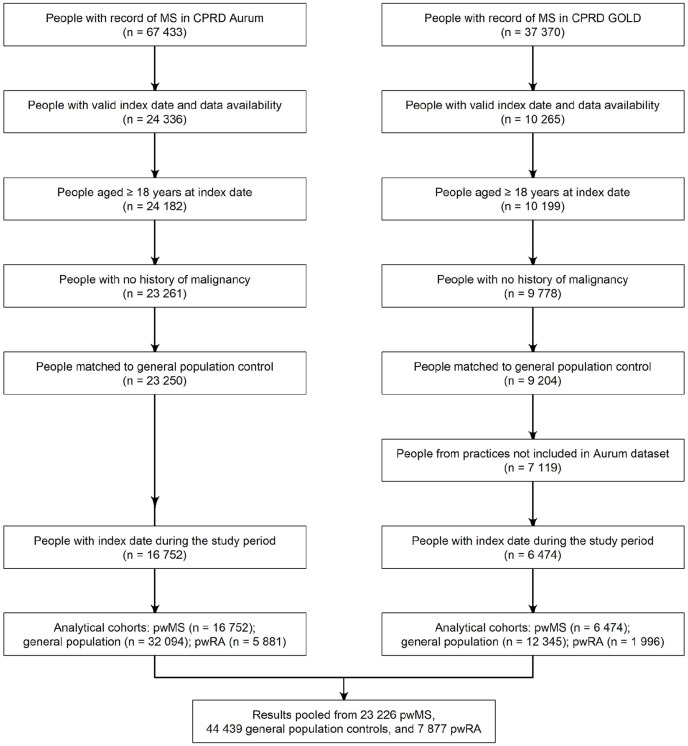
Flowchart of patient inclusion in the study. CPRD: Clinical Practice Research Datalink; pwMS: people with multiple sclerosis; pwRA: people with rheumatoid arthritis.

67,433 people had a record of MS in CPRD Aurum, and 37,370 in CPRD GOLD. Approximately 20% met the eligibility criteria: 16,752 pwMS were included from CPRD Aurum and 6474 from CPRD GOLD (Figure 2). Overall, 23,226 pwMS were included, matched to 44,439 general population controls and 7877 RA controls – not every pwMS could be matched to pwRA. The baseline characteristics were first tabulated separately for CPRD Aurum and GOLD. Given the similarity between them (Table S1), they were reported together in Table 1. PwMS were followed for median 5.9 years (IQR: 2.4–11.0), the median age was 43 years and approximately 70% of pwMS were women. The median age and proportion of women remained stable over the study period.

**Table 1.** Baseline characteristics of pwMS, matched general population and pwRA in the pooled CPRD Aurum and GOLD cohorts.

Characteristic	pwMS (*n* = 23,226)	General population (*n* = 44,439)	pwRA (*n* = 7877)
	*n* (%)	*n* (%)	*n* (%)
CPRD Aurum	16,752 (72.1)	32,094 (72.2)	5881 (74.4)
Year of index date			
2000–2005	6376 (27.5)	12,296 (27.7)	2149 (27.3)
2006–2010	5719 (24.6)	10,946 (24.6)	1865 (23.7)
2011–2015	5570 (24.0)	10,627 (23.9)	1987 (25.2)
2016–2020	5561 (23.9)	10,570 (23.8)	1876 (23.8)
Follow-up time after index date (years)			
Median [IQR]	5.9 [2.4–11.0]	6.0 [2.4–11.4]	7.0 [3.3–12.1]
Age at index date (years)			
Median [IQR]	43.0 [35.0–52.0]	43.0 [34.0–51.0]	49.0 [42.0–57.0]
Women	16,349 (70.4)	31,190 (70.2)	6429 (81.6)
History of disease (ever before)			
Chronic lung disease	3227 (13.9)	5712 (12.9)	1377 (17.5)
Diabetes mellitus	903 (3.9)	1527 (3.4)	483 (6.1)
Rheumatoid arthritis	150 (0.6)	289 (0.7)	
Kidney disease	640 (2.8)	1009 (2.3)	315 (4.0)
Inflammatory bowel disease	238 (1.0)	427 (1.0)	79 (1.0)
Psoriasis	742 (3.2)	1386 (3.1)	220 (2.8)
Cardiovascular risk factor^a^	3794 (16.3)	6567 (14.8)	1871 (23.8)
Lymphocyte or neutrophil count (previous 12 months)	9591 (41.3)	9090 (20.5)	5798 (73.6)
History of infections (previous 12 months)			
1	3831 (16.5)	6332 (14.2)	1460 (18.5)
⩾2	1513 (6.5)	2206 (5.0)	661 (8.4)
Medication prescriptions (previous 3 months)			
⩾1 corticosteroid^b^	1011 (4.4)	482 (1.1)	1000 (12.7)
⩾1 immunomodulator^c^	2227 (9.6)	3844 (8.7)	2322 (29.5)
Medication prescriptions (previous 12 months)			
Symptomatic drugs^d^			
1	5571 (24.0)	6057 (13.6)	1697 (21.5)
2	2565 (11.0)	1706 (3.8)	584 (7.4)
⩾3	1610 (6.9)	669 (1.5)	276 (3.5)
⩾1 antimicrobial	7336 (31.6)	11401 (25.7)	2826 (35.9)

CPRD: Clinical Practice Research Datalink; IQR: interquartile range; pwMS: people with multiple sclerosis; pwRA: people with rheumatoid arthritis.

a Cardiovascular risk factor: hypertension, hyperlipidaemia, myocardial infarction, stenting or coronary artery bypass, arrhythmia, valvular disease, heart murmurs, cardiomegaly and congestive heart failure.

b Corticosteroids used in the acute treatment of MS relapses.

c Immunomodulatory treatments: disease-modifying antirheumatic treatments, anti-inflammatory treatments, hydroxycarbamide, interferon products, melphalan, mercaptopurine and corticosteroids not used in the acute treatment of MS relapses.

d Symptomatic drugs: products to treat migraine, sexual dysfunction, dystonia, neuropathic pain, tremor, epilepsy, enuresis, skeletal muscle relaxants, benzodiazepines and antidepressant drugs.

### Infection rates

**Figure 3.**
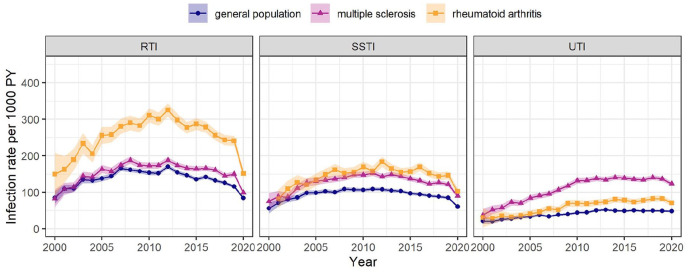
Infection rates over calendar time. Shaded area: 95% confidence interval. PY: person-year; RTI: respiratory tract infection; SSTI: skin and subcutaneous tissue infection; UTI: urinary tract infection.

The rate of infection of any of the five types was 429 per 1000 PY (95% CI: 426–433) among pwMS, 1.51-fold (95% CI: 1.49–1.52) the rate among the general population and 0.87-fold (95% CI: 0.86–0.88) the rate among pwRA (Table 2). Sepsis and GTI were hardly detected and were not studied individually. The UTI rate was most elevated among pwMS: 125 per 1000 PY (95% CI: 123–127), 2.80-fold (95% CI: 2.74–2.86) the rate among the general population and 1.85-fold (95% CI: 1.79–1.91) the rate among pwRA (Figure 3). The RTI rate was elevated among pwRA compared with pwMS. The SSTI rate was higher among pwMS than the general population, and similar to pwRA. The UTI rate increased on average 1.02-fold per year (95% CI: 1.01–1.03) among pwMS and the general population, while rates of RTI and SSTI did not increase. Time trends were examined but did not differ between age groups, gender and the presence or absence of infection ⩽ 1 year before index date.

**Table 2.** Infection rates (IRs) per 1000 person-years (PY) with 95% confidence intervals (CIs) among people with MS (pwMS), general population and people with RA (pwRA).

	IR per 1000 PY [95% CI]		
	pwMS	General population	pwRA
Any infection: GTI, RTI, SSTI, UTI or sepsis	429 [426–433]	287 [285–288]	497 [492–503]
RTI	160 [158–162]	139 [137–140]	264 [260–268]
SSTI	132 [130–134]	96 [95–97]	151 [148–154]
UTI	125 [123–127]	45 [45–46]	69 [67–71]

CI: confidence intervals; GTI: gastrointestinal tract infection; IR: infection rates; PY: person-years; pwMS: people with multiple sclerosis; pwRA: people with rheumatoid arthritis; RTI: respiratory tract infection; SSTI: skin and subcutaneous tissue infection; UTI: urinary tract infection.

Sepsis and GTI were not studied individually because they were detected at low rates.

**Figure 4.**
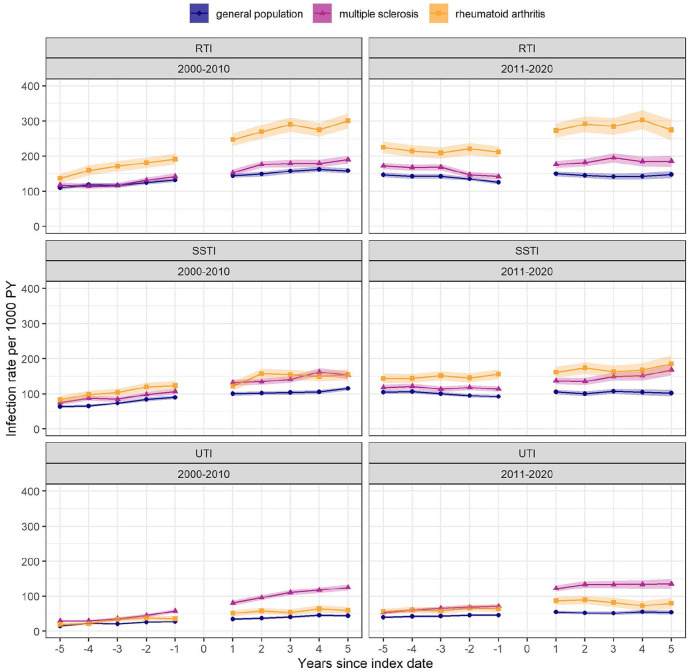
Infection rates from 5 years before to 5 years after index date, stratified by index date during 2000–2010 or 2011–2020. Results are based on approximately 70% of the study population, who had ⩾ 5 years of medical history before index date: 16,334 people with multiple sclerosis, 31,340 general population controls and 6203 people with rheumatoid arthritis. Shaded area: 95% confidence interval. PY: person-year; RTI: respiratory tract infection; SSTI: skin and subcutaneous tissue infection; UTI: urinary tract infection.

Among pwMS, the UTI rate was 2.24 (95% CI: 2.16–2.33) times as high during 5 years after the index date as during 5 years before, compared with 1.39 (95% CI: 1.33–1.44) times among the general population and 1.47 (95% CI: 1.37–1.58) times among pwRA (Figure 4). Across the three types of infection and the three cohorts, the post-index date infection rates were similar during both decades whereas pre-index date infection rates were higher during 2011–2020 than in 2000–2010.

The results were similar in the sensitivity analysis with 60-day episodes of infection instead of 30-day episodes.

### Predictors of infection

PwMS experienced median 1 (IQR: 0–3) infection of any of the five types during the 5 years after MS diagnosis, with observed maximum 32 (25 in the sensitivity analysis with 60-day episodes of infection). Characteristics associated with more infections were: infection ⩽ 12 months before diagnosis, female sex, symptomatic drug prescription ⩽ 12 months before diagnosis, presence of comorbidity, lymphocyte or neutrophil count ⩽ 12 months before diagnosis and immunomodulatory drug prescription ⩽ 3 months before diagnosis. Age at MS diagnosis ⩾ 30 years was associated with a lower infection rate. IRRs and 95% CIs of the predictor variables from the Poisson regression were similar between the two halves of the study period; the overall results are shown in Table 3. The sensitivity analysis using the Andersen–Gill model gave similar results (Table S2).

**Table 3.** Predictive factors of infection during the 5 years after MS diagnosis.

	IRR [95% CI]
Infections during 12 months before MS diagnosis	
1	1.92 [1.86–1.97]
⩾2	3.00 [2.89–3.10]
Female sex	1.48 [1.44–1.53]
Symptomatic drugs^a^ prescribed during 12 months before MS diagnosis	
1	1.22 [1.18–1.26]
2	1.48 [1.42–1.53]
⩾3	1.75 [1.67–1.82]
⩾1 comorbidity^b^ ever before MS diagnosis	1.19 [1.16–1.22]
⩾1 immunomodulatory treatment^c^ prescription during 3 months before MS diagnosis	1.15 [1.11–1.20]
⩾1 neutrophil or lymphocyte count	1.11 [1.08–1.14]
Age at MS diagnosis ⩾ 30 years	0.78 [0.75–0.81]

CI: confidence interval; IRR: infection rate ratio; MS: multiple sclerosis.

Results are based on 12,862 people with MS (pwMS) (55.4% of the included pwMS) with ⩾ 5 years of follow-up.

a Symptomatic drugs: products to treat migraine, sexual dysfunction, dystonia, neuropathic pain, tremor, epilepsy, enuresis, skeletal muscle relaxants, benzodiazepines and antidepressant drugs.

b Comorbidity: diabetes mellitus, chronic lung disease (chronic obstructive pulmonary disease and asthma), inflammatory bowel disease, RA, psoriasis, kidney disease (chronic kidney disease, nephritis, renal hypertensive disease and cystic kidney disease) and cardiovascular risk factor (hypertension, hyperlipidaemia, myocardial infarction, stenting or coronary artery bypass, arrhythmia, valvular disease, heart murmurs, cardiomegaly and congestive heart failure).

c Immunomodulatory treatments: disease-modifying antirheumatic treatments, anti-inflammatory treatments, hydroxycarbamide, interferon products, melphalan, mercaptopurine and corticosteroids not used in the acute treatment of MS relapses.

## Discussion

Over the past two decades, pwMS had a consistently higher risk of infections than the general population. Of the five types of infection studied, the UTI rate increased most notably after index date among pwMS compared with the general population and pwRA. The large variation between pwMS in infection rates after MS diagnosis signals a burden on specific pwMS who experience multiple or recurrent infections. This may be of particular concern to women, and pwMS who, during the year before MS diagnosis, experienced at least one infection or were prescribed a symptomatic drug at least once.

Overall infection rates among pwMS have remained stable over the past 20 years. The rate of any infection was 1.51 times as high in pwMS as in the general population, similar to estimates from previous studies.^6,7^ The UTI rate among pwMS was 2.80-fold the rate among the general population, which was higher than previously reported. This may be due both to the sensitive approach to UTI ascertainment in this study, as not only diagnoses but also symptoms and tests followed by antibiotic prescription were used, and to the inclusion of all episodes of UTI during follow-up rather than only the first after index date. The UTI rate increased 1.02-fold per year of the study period among pwMS and the general population, in line with findings among older (⩾65 years) people seen in the UK primary care,^16^ and increasing rates of nitrofurantoin and trimethoprim prescriptions to people aged 25–84 years in the UK.^31^

Rates of RTI, SSTI and UTI among pwMS were higher than general population already during the 5 years before index date, and the UTI rate during the 5 years after MS diagnosis was 2.24-fold the rate during the 5 years before. Previous studies have also reported increased susceptibility to infections among pwMS up to 1–5 years pre-index date,^32,33^ which may result from underlying MS disease in the prodromal phase. Both pwMS and pwRA were more susceptible to infections after index date. Possible causes of the increased infection risk among both pwMS and pwRA include the underlying disease process, physicians’ heightened awareness of infection risk and treatment: DMTs for MS, and biologic and targeted-synthetic disease-modifying antirheumatic drugs for RA. Disease-specific functional limitations may partly explain infection patterns: urinary system dysfunction due to MS disease progression may contribute specifically to the UTI rate among pwMS, while rheumatoid lung disease may contribute to the RTI rate among pwRA.

The patient characteristics, determined at the time of MS diagnosis, associated with the risk of infection after MS diagnosis did not change during the study period: recent infection, female sex, recent symptomatic drug prescription, the presence of comorbidity, recent lymphocyte or neutrophil count and recent immunomodulatory drug prescription were associated with a higher infection rate after MS diagnosis, while age ⩾ 30 years was associated with a lower infection rate. In contrast, a study investigating infection-related hospitalization among US veterans reported that increased risk was associated with male sex and higher age, but not recent immunosuppressive treatment prescription.^18^ Another study, investigating infection-related physician visits among British Columbia residents, reported a higher rate among women and current age groups below or above 40–49 years.^6^ The differences in findings may be due to differences in the study population, with the findings from this study more similar to the latter, population-based study. The lower infection rate within 5 years after MS diagnosis in people aged ⩾ 30 years at MS diagnosis may be due to differences in lifestyle. Although corticosteroid exposure has been associated with infection,^34,35^ recent corticosteroid prescription was not predictive of infection in this study. This may be due to the lack of capture of specialist prescriptions of intravenous methylprednisolone in the CPRD database.

In this multi-database study, the examination of infection rates among pwMS over the past two decades allowed us to better understand whether the changing pwMS and treatment options have changed the infection risk. The two UK GP databases resulted in a large, representative sample of pwMS over the study period. The inclusion of both general population and pwRA controls provided different insights into the elevated infection risk in pwMS: direct matching between pwMS and pwRA allowed for a comparison of infection risk and time trends in infection risk between pwMS and people with similar underlying disease course and availability of disease-modifying treatments. Another strength of the study was the interest in repeated outcomes rather than time-to-event outcomes, which highlighted the burden of multiple and recurrent infections on pwMS. One limitation of the study was that the primary care data did not capture specialist prescriptions. It was thus not possible to directly examine whether infection risk differs according to DMT use, although previous studies have examined this. Nevertheless, by looking at infection rates over the past two decades and before and after MS diagnosis, we gained insights into the effect of DMTs on infection risk at the population level and individual level. Another limitation of the study was that we could not be certain that everyone with ⩾ 1 MS record in our UK GP datasets actually had MS. We could have been more stringent in the inclusion criteria, however, we preferred sensitivity over specificity in the selection of pwMS. Any misclassification of non-pwMS as pwMS would result in an underestimation of the increased infection risk among pwMS compared with the general population. The majority (65.1%) of pwMS included had at least two MS records (CPRD Aurum: 72.6%; CPRD GOLD: 45.4%). Third, the age at MS diagnosis was slightly higher than in clinical studies, which may be due to difficulty in identifying the moment of first diagnosis in UK GP data. Fourth, it was not possible to match pwRA to pwMS in a 1:1 ratio. The included pwRA had a higher age at the index date and a larger proportion of women. More pwRA would have been included if the matching criteria had been relaxed further, but this would have harmed the comparability between pwMS and pwRA. Finally, sepsis and GTI could not be studied individually because they were hardly detected, possibly because sepsis seen in secondary care may not always be recorded in primary care and people may not always visit the GP for GTI.

Although the overall risk of infection in pwMS remained stable over the past two decades when new treatments became available, the rate of UTI increased. Rates of RTI, SSTI and UTI were already elevated before MS diagnosis, with the largest increase in the UTI rate after MS diagnosis. A number of patient characteristics at the time of MS diagnosis, most strongly recent infection, female sex and recent symptomatic drug prescription, were associated with increased risk of infection after MS diagnosis, but the infection rate varied widely between individuals. The findings of this study indicate that pwMS and clinicians should be aware of the risk of infections, as well as recurrence of infections, especially UTIs, which may have a substantial effect on the quality of life of pwMS. Further research should be performed for a better understanding of the dynamics between patient-related and treatment-related factors that affect the risk of infection from the moment of MS diagnosis onwards, to facilitate risk stratification and management of modifiable factors related to infections.

## Supplemental Material

sj-docx-1-msj-10.1177_13524585221094218 – Supplemental material for Mapping the risk of infections in patients with multiple sclerosis: A multi-database study in the United Kingdom Clinical Practice Research Datalink GOLD and Aurum Click here for additional data file. Supplemental material, sj-docx-1-msj-10.1177_13524585221094218 for Mapping the risk of infections in patients with multiple sclerosis: A multi-database study in the United Kingdom Clinical Practice Research Datalink GOLD and Aurum by Melissa WY Leung, Marloes T Bazelier, Patrick C Souverein, Bernard MJ Uitdehaag, Olaf H Klungel, Hubert GM Leufkens and Romin Pajouheshnia in Multiple Sclerosis Journal

sj-docx-2-msj-10.1177_13524585221094218 – Supplemental material for Mapping the risk of infections in patients with multiple sclerosis: A multi-database study in the United Kingdom Clinical Practice Research Datalink GOLD and Aurum Click here for additional data file. Supplemental material, sj-docx-2-msj-10.1177_13524585221094218 for Mapping the risk of infections in patients with multiple sclerosis: A multi-database study in the United Kingdom Clinical Practice Research Datalink GOLD and Aurum by Melissa WY Leung, Marloes T Bazelier, Patrick C Souverein, Bernard MJ Uitdehaag, Olaf H Klungel, Hubert GM Leufkens and Romin Pajouheshnia in Multiple Sclerosis Journal
