# Supplementary material for: Mapping the risk of infections in patients with multiple sclerosis: A multi-database study in the United Kingdom Clinical Practice Research Datalink GOLD and Aurum
Source: Mult Scler. 2022 May 14;28(11):1808–18. doi: 10.1177/13524585221094218 (PMC9442628; doi:10.1177/13524585221094218)
Supplement: sj-docx-1-msj-10.1177_13524585221094218 – Supplemental material for Mapping the risk of infections in patients with multiple sclerosis: A multi-database study in the United Kingdom Clinical Practice Research Datalink GOLD and Aurum [file sj-docx-1-msj-10.1177_13524585221094218.docx]

**Supplemental methods: outcome ascertainment**

## Gastrointestinal tract infection (GTI)

GTI was ascertained in part through a published algorithm: either (1) a definite diagnosis code, (2) the combination of codes of a GTI symptom and a GTI pathogen within 28 days was recorded as GTI ^1^, or, an addition to the algorithm, (3) a code of a GTI symptom and an antimicrobial prescription on the same day. If a GTI symptom was recorded up to 28 days prior to a definite diagnosis, the event date of the symptom was taken as the event date of the definite GTI code ^1^. The medical code list was based on two published lists ^1,2^, of which management-related codes were omitted. The product code list comprised the antimicrobial products listed in the NICE Summary of antimicrobial prescribing guidance^3^ for common GTIs.

## Respiratory tract infection (RTI)

RTI was ascertained through (1) a definite diagnosis code for RTI or (2) a code for a RTI symptom with an antimicrobial prescription or diagnosis code for acute otitis media on the same day. The antimicrobial products considered for RTI were those listed as such in the NICE Summary of antimicrobial prescribing guidance. The main interest in RTI for this study was in lower RTI and upper RTI except for otitis. Otitis externa was included as a SSTI in this study, and the products indicated for otitis externa were not considered for ascertainment of RTI. The medical code list was based on a published code list ^4^.

## Sepsis

Sepsis was ascertained through records of diagnosis codes for sepsis or septicaemia. Codes with and without mention of a specific pathogen were included ^5^.

## Skin and subcutaneous tissue infection (SSTI)

SSTI was ascertained through (1) a definite diagnosis code for SSTI or (2) a code for a SSTI symptom with an antimicrobial prescription on the same day. This algorithm was made based on a code list from a study^6^ that included all events for which flucloxacillin might be prescribed. From this list, warts were excluded, as well as terms that were found to be not specific enough for this study. The antimicrobial products considered in the ascertainment of SSTI were those indicated in the NICE Summary of antimicrobial prescribing guidance for SSTI, but also for genital tract infections, suspected dental infections, and otitis externa.

## Urinary tract infection (UTI)

UTI was ascertained using the part of a described algorithm that does not involve linked hospital data: records with a code either for a diagnosis or symptom of UTI were selected and infection was confirmed by (1) an antibiotic prescription on the same day, or (2) a urine test on the same day and an antibiotic prescription within seven days ^7^. The product code list comprised the antimicrobial products listed in the NICE Summary of antimicrobial prescribing guidance to treat common UTIs.

**References**

1. Mansfield KE, Douglas IJ, Nitsch D, et al. Acute kidney injury and infections in patients taking antihypertensive drugs: a self-controlled case series analysis. *Clin Epidemiol* 2018; 10: 187–202.

2. Verstraeten T, Cattaert T, Harris J, et al. Estimating the Burden of Medically Attended Norovirus Gastroenteritis: Modeling Linked Primary Care and Hospitalization Datasets. *J Infect Dis* 2017; 216: 957–965.

3. Antimicrobial prescribing guidelines | NICE guidance | Our programmes | What we do | About. *NICE*, https://www.nice.org.uk/about/what-we-do/our-programmes/nice-guidance/antimicrobial-prescribing-guidelines (accessed 31 March 2020).

4. Palin V, Mölter A, Belmonte M, et al. Antibiotic prescribing for common infections in UK general practice: variability and drivers. *J Antimicrob Chemother* 2019; 74: 2440–2450.

5. Gharbi M, Drysdale JH, Lishman H, et al. Antibiotic management of urinary tract infection in elderly patients in primary care and its association with bloodstream infections and all cause mortality: population based cohort study. *BMJ* 2019; 364: l525.

6. Francis NA, Hood K, Lyons R, et al. Understanding flucloxacillin prescribing trends and treatment non-response in UK primary care: a Clinical Practice Research Datalink (CPRD) study. *J Antimicrob Chemother* 2016; 71: 2037–2046.

7. Ahmed H, Farewell D, Jones HM, et al. Incidence and antibiotic prescribing for clinically diagnosed urinary tract infection in older adults in UK primary care, 2004-2014. *PLOS ONE* 2018; 13: e0190521.
