# Supplementary material for: Mapping the risk of infections in patients with multiple sclerosis: A multi-database study in the United Kingdom Clinical Practice Research Datalink GOLD and Aurum
Source: Mult Scler. 2022 May 14;28(11):1808–18. doi: 10.1177/13524585221094218 (PMC9442628; doi:10.1177/13524585221094218)
Supplement: sj-docx-2-msj-10.1177_13524585221094218 – Supplemental material for Mapping the risk of infections in patients with multiple sclerosis: A multi-database study in the United Kingdom Clinical Practice Research Datalink GOLD and Aurum [file sj-docx-2-msj-10.1177_13524585221094218.docx]

**Table S1** Baseline characteristics of people with multiple sclerosis (pwMS), matched general population, and people with rheumatoid arthritis (pwRA) per database. CPRD: Clinical Practice Research Datalink; IQR: interquartile range. P-values: results of the chi-squared test for categorical variables and Kruskal-Wallis rank sum test for the continuous variables.

|  | **pwMS** | | | **general population** | | | **pwRA** | | |
| --- | --- | --- | --- | --- | --- | --- | --- | --- | --- |
|  | **CPRD Aurum**  **(n=16752)** | **CPRD GOLD**  **(n=6474)** | *p-value* | **CPRD Aurum**  **(n=32094)** | **CPRD GOLD**  **(n=12345)** | *p-value* | **CPRD Aurum**  **(n=5881)** | **CPRD GOLD**  **(n=1996)** | *p-value* |
| Characteristic | n (%) | n (%) |  | n (%) | n (%) |  | n (%) | n (%) |  |
| **Year of index date** | *<0.01* | | | *<0.001* | | | *<0.001* | | |
| 2000-2005 | 4855 (29.0) | 1521 (23.5) |  | 9378 (29.2) | 2918 (23.6) |  | 1649 (28.0) | 500 (25.1) |  |
| 2006-2010 | 3849 (23.0) | 1870 (28.9) |  | 7387 (23.0) | 3559 (28.8) |  | 1296 (22.0) | 569 (28.5) |  |
| 2011-2015 | 3877 (23.1) | 1693 (26.2) |  | 7403 (23.1) | 3224 (26.1) |  | 1462 (24.9) | 525 (26.3) |  |
| 2016-2020 | 4171 (24.9) | 1390 (21.5) |  | 7926 (24.7) | 2644 (21.4) |  | 1474 (25.1) | 402 (20.1) |  |
| **Follow-up time after index date (years)** | | | | | | | | | |
| Median [IQR] | 6.0 [2.5-11.5] | 5.4 [2.4-9.8] | *<0.001* | 6.1 [2.5-11.9] | 5.6 [2.4-10.1] | *<0.001* | 7.2 [3.4-12.6] | 6.6 [3.2-10.5] | *<0.001* |
| **Age at index date (years)** | | | | | | | | | |
| Median [IQR] | 43.0 [35.0-52.0] | 43.0 [34.0-52.0] | *0.549* | 43.0 [34.0-51.0] | 43.0 [34.0-51.0] | *0.432* | 49.0 [42.0-57.0] | 49.0 [42.0-56.0] | *0.446* |
| **Women** | 11766 (70.2) | 8714 (70.6) | *0.416* | 22476 (70.0) | 8714 (70.6) | *0.256* | 4802 (81.7) | 1627 (81.5) | *0.916* |
| **History of disease (ever before)** | | | | | | | | | |
| Chronic lung disease | 2364 (14.1) | 863 (13.3) | *0.128* | 4069 (12.7) | 1643 (13.3) | *0.078* | 1035 (17.6) | 342 (17.1) | *0.661* |
| Diabetes mellitus | 646 (3.9) | 257 (4.0) | *0.716* | 1120 (3.5) | 407 (3.3) | *0.332* | 359 (6.1) | 124 (6.2) | *0.905* |
| Rheumatoid arthritis | 112 (0.7) | 38 (0.6) | *0.545* | 216 (0.7) | 73 (0.6) | *0.371* |  |  |  |
| Kidney disease | 466 (2.8) | 174 (2.7) | *0.728* | 743 (2.3) | 266 (2.2) | *0.327* | 237 (4.0) | 78 (3.9) | *0.861* |
| Inflammatory bowel disease | 165 (1.0) | 73 (1.1) | *0.371* | 293 (0.9) | 134 (1.1) | *0.106* | 69 (1.2) | 10 (0.5) | *0.013* |
| Psoriasis | 541 (3.2) | 201 (3.1) | *0.658* | 944 (2.9) | 442 (3.6) | *0.001* | 170 (2.9) | 50 (2.5) | *0.409* |
| Cardiovascular risk factor^a^ | 2754 (16.4) | 1040 (16.1) | *0.500* | 4725 (14.7) | 1842 (14.9) | *0.608* | 1418 (24.1) | 453 (22.7) | *0.210* |
| **Lymphocyte or neutrophil count (previous 12 months)** | 7115 (42.5) | 2476 (38.2) | *<0.001* | 6674 (20.8) | 2416 (19.6) | *0.004* | 4379 (74.5) | 1419 (71.1) | *0.003* |
| **History of infections (previous 12 months)** | *0.090* | | | *0.522* | | | *0.329* | | |
| 1 | 2804 (16.7) | 1027 (15.9) |  | 4543 (14.2) | 1789 (14.5) |  | 1069 (18.2) | 391 (19.6) |  |
| ≥ 2 | 1113 (6.6) | 400 (6.2) |  | 1609 (5.0) | 597 (4.8) |  | 501 (8.5) | 160 (8.0) |  |
| **Medication prescriptions (previous 3 months)** | | | | | | | | | |
| **≥ 1 corticosteroid^b^** | 647 (3.9) | 364 (5.6) | *<0.001* | 339 (1.1) | 143 (1.2) | *0.379* | 739 (12.6) | 261 (13.1) | *0.580* |
| **≥ 1 immunomodulator^c^** | 1538 (9.2) | 689 (10.6) | *0.001* | 2617 (8.2) | 1227 (9.9) | *<0.001* | 1703 (29.0) | 619 (31.0) | *0.087* |
| **Medication prescriptions (previous 12 months)** | | | | | | | | | |
| **Symptomatic drugs^d^** | *<0.001* | | | *<0.001* | | | *0.001* | | |
| 1 | 3995 (23.8) | 1576 (24.3) |  | 4203 (13.1) | 1854 (15.0) |  | 1233 (21.0) | 464 (23.2) |  |
| 2 | 1778 (10.6) | 787 (12.2) |  | 1148 (3.6) | 558 (4.5) |  | 411 (7.0) | 173 (8.7) |  |
| ≥ 3 | 1058 (6.3) | 552 (8.5) |  | 401 (1.2) | 268 (2.2) |  | 194 (3.3) | 82 (4.1) |  |
| **≥ 1 antimicrobial** | 5038 (30.1) | 2298 (35.5) | *<0.001* | 7752 (24.2) | 3649 (29.6) | *<0.001* | 2010 (34.2) | 816 (40.9) | *<0.001* |

^a^ Cardiovascular risk factor: hypertension, hyperlipidaemia, myocardial infarction, stenting or coronary artery bypass, arrhythmia, valvular disease, heart murmurs, cardiomegaly, and congestive heart failure.

^b^ Corticosteroids used in the acute treatment of MS relapses.

^c^ Immunomodulatory treatments: disease-modifying antirheumatic treatments, anti-inflammatory treatments, hydroxycarbamide, interferon products, melphalan, mercaptopurine, and corticosteroids not used in the acute treatment of MS relapses.

^d^ Symptomatic drugs: products to treat migraine, sexual dysfunction, dystonia, neuropathic pain, tremor, epilepsy, enuresis, skeletal muscle relaxants, benzodiazepines, and antidepressant drugs.

**Table S2** Predictive factors of infection during the five years after multiple sclerosis (MS) diagnosis. Poisson regression model: data from 12862 people with MS (pwMS) (55.4% of the included pwMS) with ≥5 years of follow-up; Andersen-Gill model: data from all 23226 pwMS included. IRR: infection rate ratio; HR: hazard ratio; CI: confidence interval.

|  | **Poisson regression model**  **IRR [95% CI]** | **Andersen-Gill model**  **HR [95% CI]** |
| --- | --- | --- |
| **Infections during 12 months before MS diagnosis** |  |  |
| **1** | 1.92 [1.86-1.97] | 1.70 [1.63-1.77] |
| **≥ 2** | 3.00 [2.89-3.10] | 2.77 [2.61-2.93] |
| **Female sex** | 1.48 [1.44-1.53] | 1.37 [1.32-1.42] |
| **Symptomatic drugs^a^ prescribed during 12 months before MS diagnosis** |  |  |
| **1** | 1.22 [1.18-1.26] | 1.26 [1.21-1.31] |
| **2** | 1.48 [1.42-1.53] | 1.48 [1.41-1.56] |
| **≥ 3** | 1.75 [1.67-1.82] | 1.79 [1.68-1.91] |
| **≥ 1 comorbidity^b^** | 1.19 [1.16-1.22] | 1.21 [1.17-1.25] |
| **≥ 1 immunomodulatory treatment^c^ prescription during 3 months before MS diagnosis** | 1.15 [1.11-1.20] | 1.23 [1.16-1.29] |
| **≥ 1 neutrophil or lymphocyte count** | 1.11 [1.08-1.14] | 1.09 [1.06-1.13] |
| **Age at MS diagnosis ≥30 years** | 0.78 [0.75-0.81] | 0.85 [0.81-0.89] |

^a^ Symptomatic drugs: products to treat migraine, sexual dysfunction, dystonia, neuropathic pain, tremor, epilepsy, enuresis, skeletal muscle relaxants, benzodiazepines, and antidepressant drugs.

^b^ Comorbidity: diabetes mellitus, chronic lung disease (chronic obstructive pulmonary disease and asthma), inflammatory bowel disease, RA, psoriasis, kidney disease (chronic kidney disease, nephritis, renal hypertensive disease, and cystic kidney disease), and cardiovascular risk factor (hypertension, hyperlipidaemia, myocardial infarction, stenting or coronary artery bypass, arrhythmia, valvular disease, heart murmurs, cardiomegaly, and congestive heart failure).

^c^ Immunomodulatory treatments: disease-modifying antirheumatic treatments, anti-inflammatory treatments, hydroxycarbamide, interferon products, melphalan, mercaptopurine, and corticosteroids not used in the acute treatment of MS relapses.
